# Use *ggbreak* to Effectively Utilize Plotting Space to Deal With Large Datasets and Outliers

**DOI:** 10.3389/fgene.2021.774846

**Published:** 2021-11-02

**Authors:** Shuangbin Xu, Meijun Chen, Tingze Feng, Li Zhan, Lang Zhou, Guangchuang Yu

**Affiliations:** Department of Bioinformatics, School of Basic Medical Sciences, Southern Medical University, Guangzhou, China

**Keywords:** axis break, gap plot, long sequential data, outlier, ggplot2

## Abstract

With the rapid increase of large-scale datasets, biomedical data visualization is facing challenges. The data may be large, have different orders of magnitude, contain extreme values, and the data distribution is not clear. Here we present an R package *ggbreak* that allows users to create broken axes using *ggplot2* syntax. It can effectively use the plotting area to deal with large datasets (especially for long sequential data), data with different magnitudes, and contain outliers. The *ggbreak* package increases the available visual space for a better presentation of the data and detailed annotation, thus improves our ability to interpret the data. The *ggbreak* package is fully compatible with *ggplot2* and it is easy to superpose additional layers and applies scale and theme to adjust the plot using the *ggplot2* syntax. The *ggbreak* package is open-source software released under the Artistic-2.0 license, and it is freely available on CRAN (https://CRAN.R-project.org/package=ggbreak) and Github (https://github.com/YuLab-SMU/ggbreak).

## Introduction

Many visualization methods would not be able to display a graph on a print page and this limits the publication of these results. There are several reasons. For example, the amount of data is large, the data contains outliers and squeezes the main part of the graph or both. As the volume and complexity of biomedical data are growing rapidly (O’Donoghue et al., 2018), circular graphs such as chord diagrams, sunburst diagrams, and circular phylograms, are becoming popular to save space for big data applications. However, not all horizontal methods have corresponding circular counterparts. Moreover, a circular graph also has its limitations. Compared with a horizontal chart, a circular graph is not intuitive and not easy to compare. One of the approaches to explore a large dataset is to split the data into several rows of graphs, especially for long sequences of data (e.g., time-series plot). Splitting a graph into multiple rows helps to improve the identification of data trends and patterns.

Outliers are unusual values that lie outside the overall pattern of distribution. It’s bad practice to simply exclude outlier data points since they are not always due to experimental errors or instrument errors. Outliers can be legitimate observations and could represent significant scientific effects. The identification of meaningful outliers can often lead to unexpected findings. Many analytical methods are looking for outliers. Such as differentially expressed gene detection, genome-wide association studies. Visualizing data with outliers can be challenging as the graph will be stretched or squeezed by the outliers. To overcome this issue, data transformation methods, such as log transformation, are often used to transform skewed data. Nonetheless, the transformation should be motivated by the data type. The normal distribution is widely used in biomedical research studies to model continuous outcomes and the log transformation is the most popular method that was used to reduce the skewness of the distribution. A previous study showed that log transformation would introduce new problems that are even more difficult to deal with (Feng et al., 2014). Applying log-transformation to data sets that are not log-normal distributed does not reduce skewness. If we are looking for outliers in our data, a process like a log transformation would de-emphasize them (Metcalf and Casey, 2016). Furthermore, log-transformed data shares little in common with the original data. Some plot patterns like boxplots have been implemented to solve the visualization problem of outliers that still can’t meet the requirement (Williamson et al., 1989). Broken axes have become a common feature of graphs in biomedical studies and also other research areas. Breaking the axis can simplify the outlier visualization, improve aesthetics, and save space (Amos and MedImmune, 2015). Advantages include applying to different distributions and preserving the original data scale, and thus more easy to convey the difference and variation between the low and high groups.

Displaying a plot with a gapped axis (i.e., missing range on one axis) is often used for the visualization of highly skewed data. When the bulk of the values get squeezed into a smaller region of the plot due to outliers, the gapped axis allows the plot to eliminate the open space between the outliers and the other data. Thus both data can be presented on the graph clearly. The R programming language has become one of the most popular tools for biomedical data visualization. However, creating gap plots is not well supported in R. The *plotrix* package provides *gap.plot()*, *gap.barplot()* and *gap.boxplot()* functions (Lemon, 2006), and the *gg.gap* package provides *gg.gap()* function to draw gap plots in base graphics and ggplot2 respectively. Unfortunately, these functions do not support overlay graphic layers after creating a gapped axis. Allowing further annotation on the graph is quite important because before the gapped plot is created, the graph is stretched or squeezed and it is not easy to add an annotation at the exact position. Moreover, in addition to gap plot, axis break has other applications, including displaying long sequence data in multiple rows, splitting a graph into multiple slices to zoom in and out to help interpretation of selected parts. These features are not implemented in R. To fill these gaps, we developed an R package, *ggbreak*, for creating an elegant axis break based on the grammar of graphic syntax implemented in *ggplot2*. This package provides a better solution to set axis break and can be widely applied in tailored visualization for various types of plots and data.

## Description

### Overview of the *ggbreak* Package

The *ggbreak* package was developed with the merits of *ggplot2* which is intuitive and flexible for data visualization (Wickham, 2009). The *ggbreak* package provides several scale functions including *scale_x_break()*, *scale_y_break()*, *scale_x_cut()*, *scale_y_cut()* and *scale_wrap()* to set axis break of *ggplot2* graphics (Table 1). The *scale_x_break()* and *scale_y_break()* functions create a gap plot with one or multiple missing ranges and allow users to adjust the relative width or height of plot slices (*i.e.*, zoom in or zoom out different parts of the plot). The *ticklabels* parameter can be used to specify customized axis labels of the plot slices. The *scale_x_cut()* and *scale_y_cut()* functions cut the plot into multiple slices to allow zoom in or zoom out of selected parts (e.g., allocating more space to display differentially expressed genes with labels in a volcano plot). The *scale_wrap()* function splits a plot over multiple rows to make the plot with a long *x*-axis (*e.g.*, time-series graphics) easier to read. The *ggbreak* package is fully compatible with *ggplot2*. After wrapping, breaking, and cutting axes of a plot, users are free to superpose multiple geometric layers from different data sources and apply theme and other scale settings. Plots created by *ggbreak* are compatible with *patchwork* and *aplot* to produce a composite plot.

**TABLE 1:** Major functions of *ggbreak*.

Function	Description
scale_wrap	Wraps a ‘gg’ plot over multiple rows
scale_x_break	Set an *x*-axis break point
scale_y_break	Set a *y*-axis break point
scale_x_cut	Set an *x*-axis divide point
scale_y_cut	Set a *y*-axis divide point

### Case Study

#### Example 1: Automatically Wrap Plot with Long *x*-Axis Scale

**FIGURE 1:**
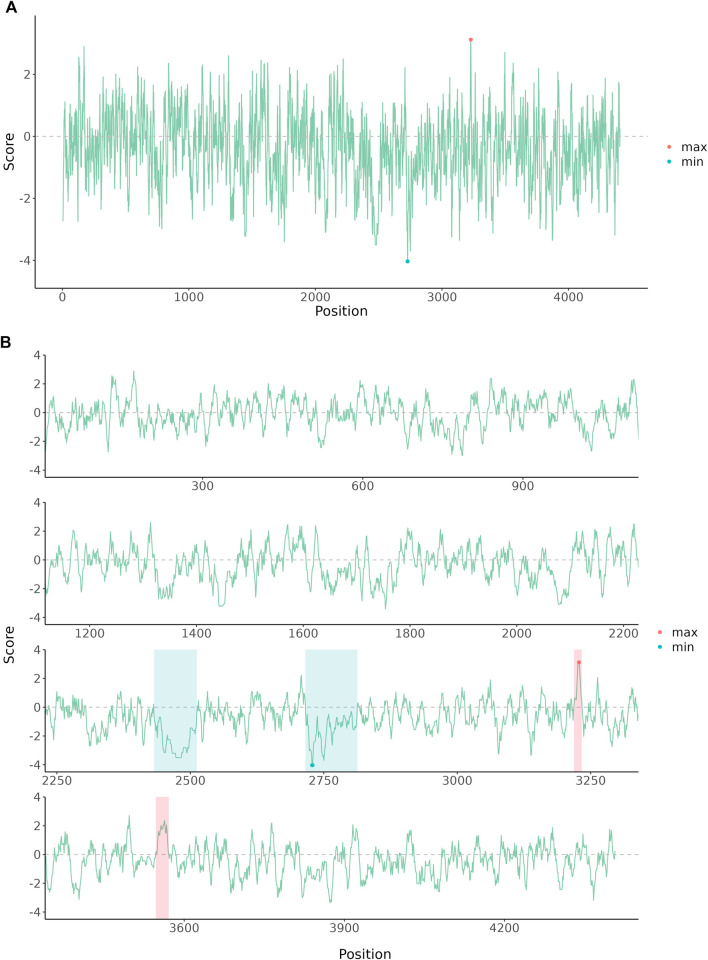
The amino acid hydrophobicity or hydrophilicity scales of human E3 ubiquitin-protein ligase HUWE1 Chain A. The protein sequence was downloaded from the NCBI database (PDB: 7MWE_A). **(A)** The original plot of the amino acid scales. **(B)** The amino acid scales were wrapped into four rows and further annotated to highlight hydrophilicity and hydrophobicity regions.

Graphs for long sequence data usually are squeezed and difficult to interpret due to the limited size of a print page. Wrapping plot for large-scale data into multiple panels helps users to identify sequential patterns. Here we provided an example to demonstrate the wrap plot implemented in *ggbreak*. The amino acid scales are numeric features of amino acids that are used to analyze protein sequences. Especially hydrophilicity/hydrophobicity scales are frequently used to characterize protein structures. Results of hydrophilicity/hydrophobicity scales usual are presented as a line chart. For long protein sequences, the line would be crowded in the graph because of highly divergent trends of the hydrophilicity/hydrophobicity scales. The protein sequence was downloaded from the NCBI database (PDB: 7MWE_A) and then the hydrophilicity/hydrophobicity scales were analyzed using *Expasy-ProtScale* with default parameters (Gasteiger et al., 2003). As showed in Figure 1A, the line is highly squeezed which makes it difficult for interpreting and understanding the sequential patterns. Splitting the plot into four rows makes the trends more clear to read (Figure 1B). The hydrophilicity regions and hydrophobicity regions are easier to identify through the whole sequence. Highlighted regions showed a clear division of hydrophilicity regions and hydrophobicity regions.

#### Example 2: Shrank Outlier Long Branch of a Phylogenetic Tree

**FIGURE 2:**
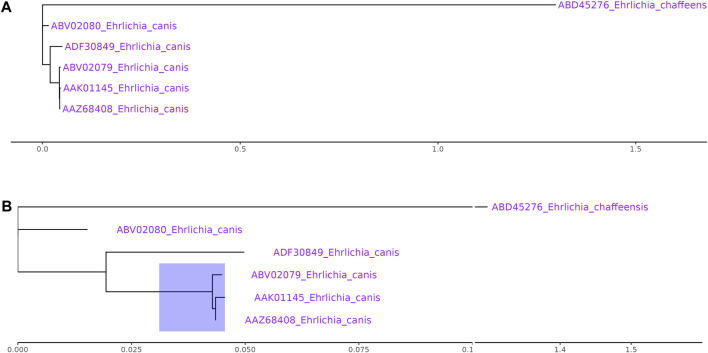
Phylogenetic tree with outlier long branch. **(A)** The original plot fails to present the detailed topological structure of the main group. **(B)** The tree with a gapped axis to shrank the outlier long branch improves the readability of the main group. All sequences were collected from NCBI database (*Ehrlichia chaffeens*: ABD45276; *Ehrlichia canis*:ABV02080, ADF30849, ABV02079, AAK01145, AAZ68408).

Data outliers may have their biological meanings and are important in the studies. It is not appropriate to simply discard outliers in these scenarios. Data transformation de-emphasizes the outliers and is not always appropriate. Using broken axes is much simple and convenient for outlier data visualization since it preserves the original scale and works for known and unknown data distributions. A phylogenetic tree is widely used to model evolutionary relationships. An outgroup is usually employed to root the unrooted tree. As the outgroup is dissimilar to the main group, it may be placed on the outlier long branch. Phylogenetic tree with outlier long branch is difficult to display well as the main group will be squeezed into a smaller space (Figure 2A). The example data were collected from the NCBI database (Huang et al., 2010). After shrinking the outlier long branch using *ggbreak*, the detailed topological structure of the highlighted region can be displayed (Figure 2B).

#### Example 3: Cut Manhattan Plot to Create More Space for Annotation

**FIGURE 3:**
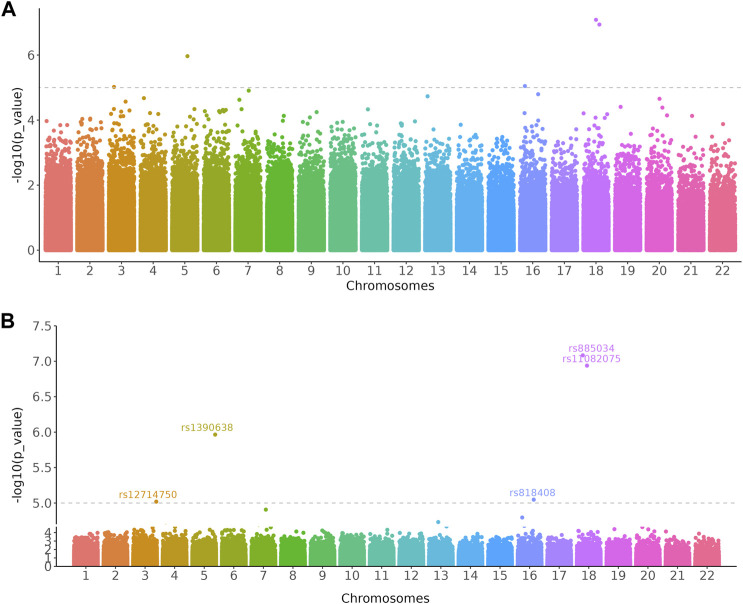
Manhattan plots showed loci significantly associated with the relative abundance of gut bacterial diversity using Chao1 index from 541 East Asians. **(A)** Original Manhattan plot. **(B)** Manhattan plot with significant region zoomed in. The dashed line represents a genome-wide suggestive level (*p* = 1 × 10^–5^). The GWAS data was downloaded from the GWAS Catalog database (Study accession: GCST90007012).

Data presented on the graph is not equally important and researchers may want to zoom in on specific regions that are significant to the results. For instance, biologists want to focus on the differentially expressed genes (DEGs) of transcriptome data on a volcano plot. The *scale_x_cut()* and *scale_y_cut()* functions implemented in *ggbreak* allow users to zoom in significant regions of a plot. Here we use the Manhattan plot to demonstrate this feature. Manhattan plot is a kind of scatter plot and is commonly used in genome-wide association studies (GWAS) to display significant single nucleotide polymorphisms (SNPs). Researchers usually focus on the upper part of the graph that displays many significant results. It is difficult to label these significant results because these labels tend to overlap in a limited space and make it difficult to read. With the *scale_y_cut()* function, it is easy to zoom in on significant regions. The example data was collected from the GWAS Catalog database (Study accession: GCST90007012) (Buniello et al., 2019; Ishida et al., 2020). The lower part was zoomed out to save space for further annotation of the upper part and thus making it easier to highlight and interpret significant results (Figure 3).

#### Example 4: Display Discontinuous Axis on a Bar Chart

**FIGURE 4:**
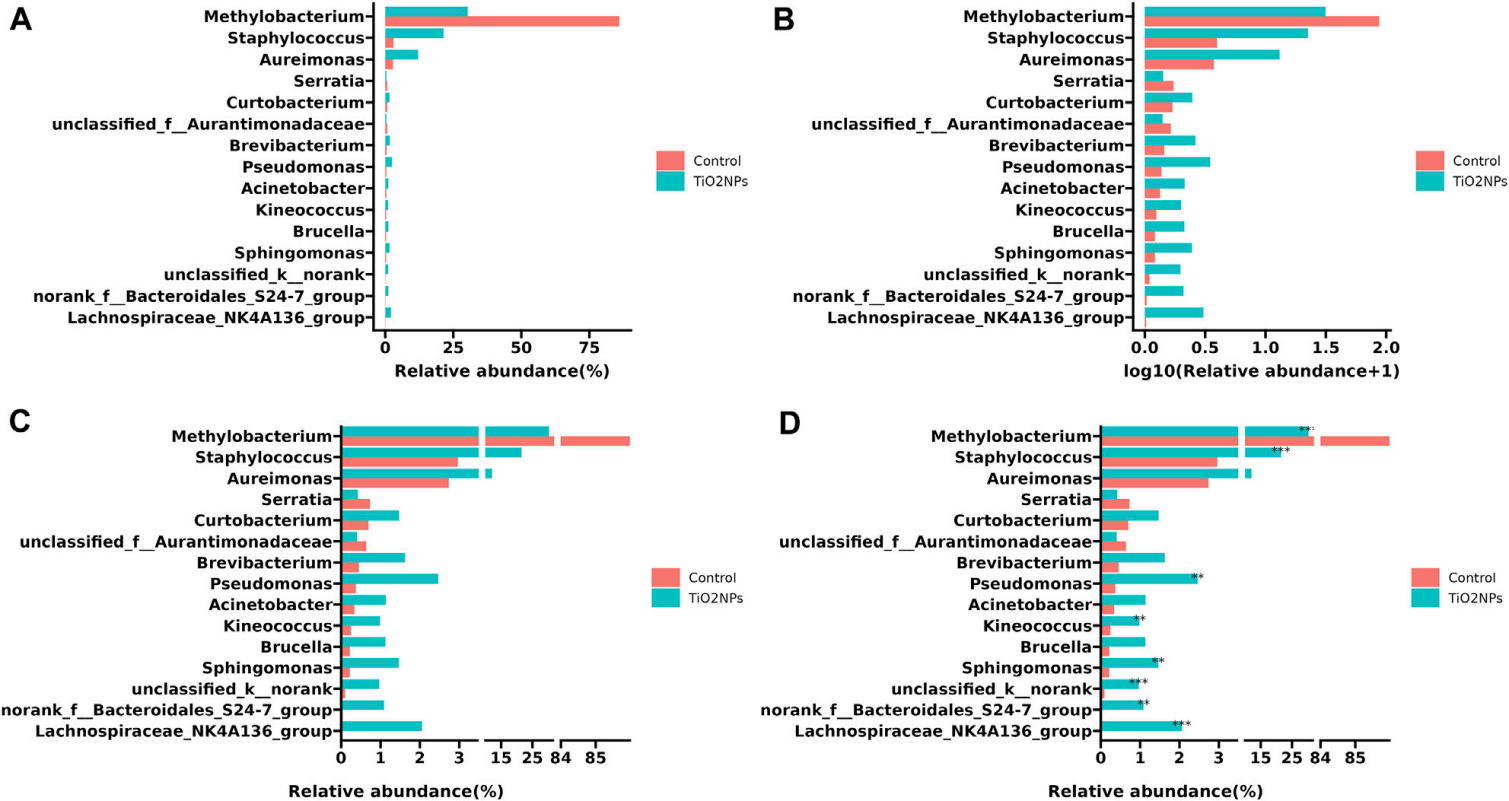
Visualizing relative microbe abundances using bar charts to show the top 15 significant genera between two groups. **(A)** Ordinary bar chart that is difficult to deal with outliers. **(B)** Bar chart with log transformation on the *x*-axis to remove skewness. **(C)** Bar chart with a gapped axis that can effectively deal with data of different magnitude. **(D)**. Superposed significant level layer to the gapped plot **(C)**. The example data was collected from a paper published in 2020 (Li et al., 2020).

Since data have different magnitudes, visualizing data with gaps (missing ranges) is frequently used in biomedicine studies, especially for bar charts. For example, in metagenomics research, microbe abundance often has different orders of magnitude, with dominant microbes account for the major proportion, while other minor catalogs only account for a small fraction. To show microbe abundances properly, a common way is to create gaps in an axis. The data used in the following example was obtained from a published paper that has described relative abundances of the top 15 genera showing significant differences among samples from the TiO2NPs-treated group and Control group (Li et al., 2020). The value of *Methylobacterium* in the Control group is much higher than other observations (Figure 4A). Log transformation is a widely used method to reduce the skewness of the data (Figure 4B). However, the transformed data and the original data are not on the same scale, which will affect the interpretation of the data. Inserting two gaps in the axis makes it much more visible for other small observations. So that the relative abundance pattern of microbes is clear at a glance (Figure 4C). In addition, the gapped plot shares similar features with the log-transformed one and the result is intuitive and easy to interpret. Unlike log-transformation, a gapped plot can be applied to all data. Furthermore, it is easier to annotate the gapped plot (e.g., superpose labels of significant level) since the scale of the value is the same as the original data (Figure 4D).

## Conclusion

Gapped axis is quite regularly used in biomedical data visualization, but it is not well implemented in R. Here, we provide a fully functional tool, *ggbreak*, which can easily use the *ggplot2* grammar of graphics syntax to create a gapped axis. The output is still a *ggplot* object that can be further superposed annotation layers and customized by applying scale and theme settings. Unlike other software designed mainly for bar charts, *ggbreak* can be applied to all graphics generated by *ggplot2*. Moreover, *ggbreak* expands the usage of broken axes by applying it to wrap long sequential data and zoom in on important regions. The usage of axes breaks should depend on the data type. Inserting axis breaks appropriately would make the graphs much more readable and improve our ability to interpret the data.

## Code Availability

**FIGURE 5:**
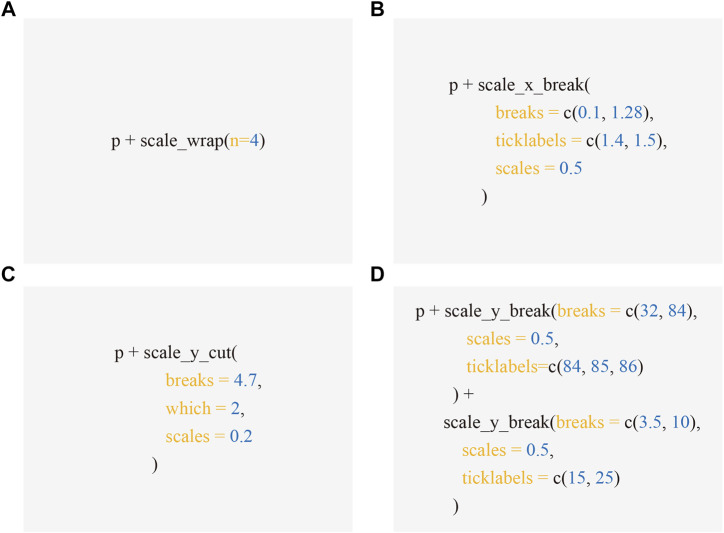
Code excerpts to produce Figures 1-4. Applying scale functions implemented in ggbreak allows creating axis break to produced Figure 1B **(A)**, 2B **(B)**, 3B **(C)**, and 4C **(D)** respectively from the subplot A, which is represented by the p object - a graphic object produced by ggplot2.

The ggbreak package is freely available on CRAN (https://CRAN.R-project.org/package=ggbreak). The excerpts of the source code that produced Figures 1-4 are presented in Figure 5. The complete code is available in Supplemental Material. R markdown file and data sets used to generate the Supplemental File are available on Github (https://github.com/YuLab-SMU/supplemental-ggbreak).

## Data Availability

The original contributions presented in the study are included in the article/Supplementary Material, further inquiries can be directed to the corresponding author.
