# Supplementary material for: Use ggbreak to Effectively Utilize Plotting Space to Deal With Large Datasets and Outliers
Source: Front Genet. 2021 Nov 2;12:774846. doi: 10.3389/fgene.2021.774846 (PMC8593043; doi:10.3389/fgene.2021.774846)
Supplement: Supplementary file 1 [file DataSheet1.PDF]

Supplemental File of

Use ggbreak to effectively utilize plotting space to deal with large datasets and outliers

Shuangbin Xu#, Meijun Chen#, Tingze Feng, Li Zhan, Lang Zhou, Guangchuang Yu*

# These authors contributed equally to this work * Correspondence: Guangchuang Yu <gcyu1@smu.edu.cn>

1 Example 1: Automatically wrap plot with long x-axis scale

```
library(ggplot2)
library(ggbreak)
library(aplot)

# import output data from Protscale
data <- read.table(file = "../data/7MWE_A.csv", sep = ",", header = F, fill = T)
colnames(data) <- c("Position","Score")
head(data)
```

```
##   Position  Score
## 1        5 -2.744
## 2        6 -2.533
## 3        7 -1.944
## 4        8 -1.600
## 5        9 -0.967
## 6       10 -0.967
```

```
data2 <- data.frame(
             Position = c(2728, 2729, 3228),
             Score = c(-4.033, -4.033, 3.122),
             Type = c("min", "min", "max")
         )

p <- ggplot() +
     geom_line(
         data = data,
         mapping = aes(
             x = Position,
             y = Score
         ),
         color = "#83cbac"
     ) +
     geom_point(
         data = data2,
         mapping = aes(
             x = Position,
             y = Score,
             color = Type
         )
     ) +
     geom_hline(
         yintercept = 0,
         color = "grey",
         linetype = "dashed"
     ) +
     expand_limits(y = c(-4.5, 3.5)) +
     theme_classic() +
     theme(
         axis.text = element_text(size = 14),
         axis.title = element_text(size = 16),
         legend.title = element_blank(),
```

```
        legend.text = element_text(size = 14)
    )
# scale_wrap of ggbreak can wrap the ggplot object to multiple
# rows to make it easier to read, the n parameter means the
# number of subplot pieces.
p1 <- p +
      scale_wrap(n=4) +
      annotate("rect", xmin=2432, xmax=2512, ymin=-4.5, ymax=4, fill='#57c3c2', alpha = 0.2) +
      annotate("rect", xmin=2716, xmax=2813, ymin=-4.5, ymax=4, fill='#57c3c2', alpha = 0.2) +
      annotate("rect", xmin=3219, xmax=3233, ymin=-4.5, ymax=4, fill='#ef475d', alpha = 0.2) +
      annotate("rect", xmin=3547, xmax=3571, ymin=-4.5, ymax=4, fill='#ef475d', alpha = 0.2)

plot_list(p, p1, ncol=1, tag_levels = 'A', tag_size = rel(2), heights=c(1, 2))
```

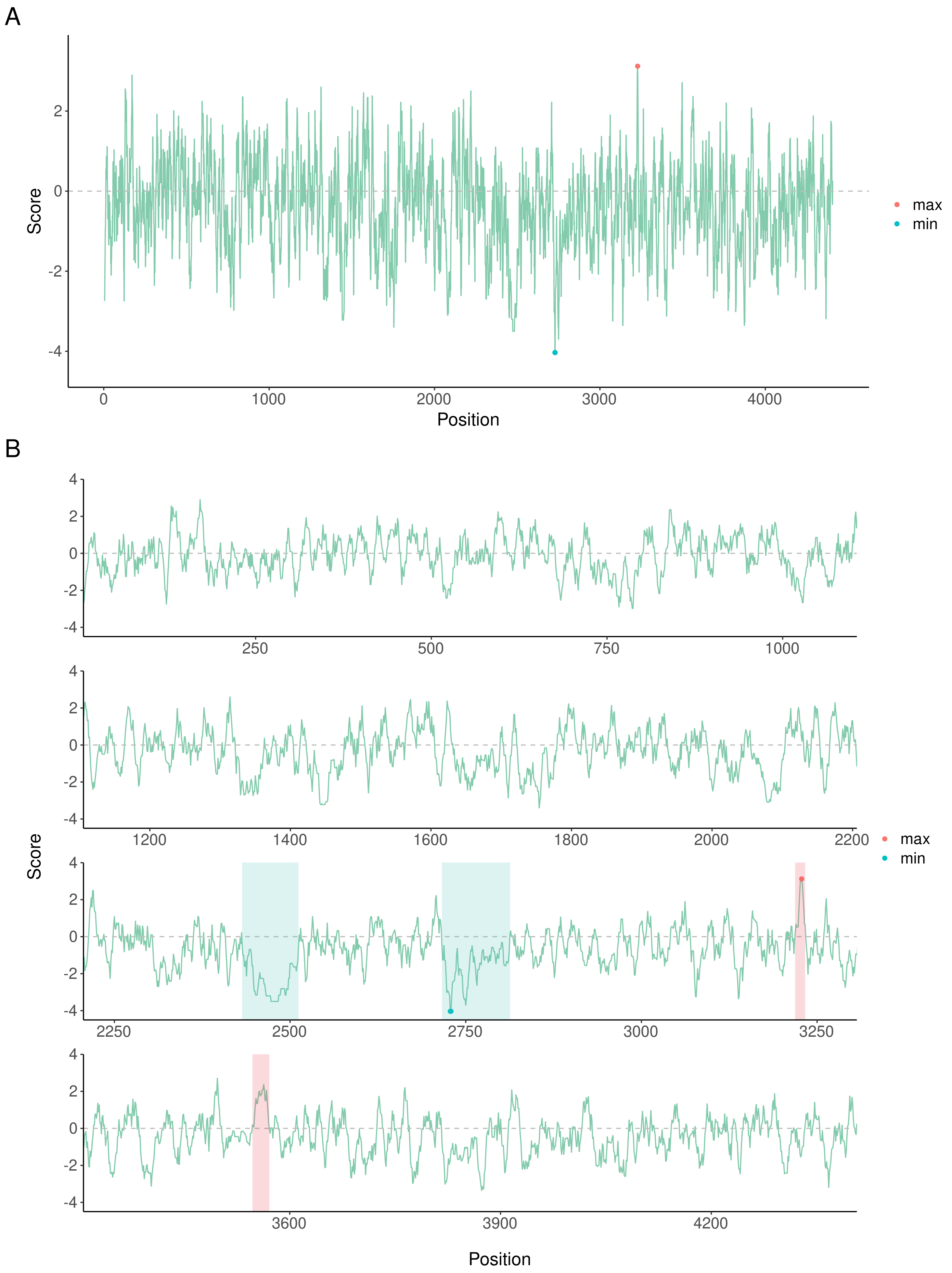
A
Score
2
0
-2
-4
0
1000
2000
3000
4000
Position
max
min
B
Score
4
2
0
-2
-4
250
500
750
1000
1200
1400
1600
1800
2000
2200
2250
2500
2750
3000
3250
3600
3900
4200
Position

2 Example 2: Shrank outlier branch of a phylogenetic tree

```
library("ggtree")
library("treeio")
library("ggbreak")
library("aplot")
library("ggplot2")

treedata <- read.newick(file = "../data/gp200.nwk")
p <- ggtree(
         treedata,
         ladderize=FALSE
     ) +
     expand_limits(x = c(0, 1.6), y = c(0, 7)) +
     geom_tiplab(size = 4, color = "purple")  +
     theme_tree2()
# In the example, the scale_x_break of ggbreak is used to set an x axis break point for the
# ggtree plot, breaks parameter means the break point. Here, the plot will be split two subplots
# ticklabels parameter means the x axis ticks labels of subplot.
# scales parameter means the relative width of subplot to the first subplot (left subplot)
# in the example
p1 <- p +
      scale_x_break(
          breaks = c(0.1, 1.28),
          ticklabels = c(1.4, 1.5),
          scales = 0.5
      ) +
      geom_highlight(
          node = 9,
          fill = "blue",
          alpha = 0.3
      ) +
      theme(
          plot.margin = margin(
              t = 2,
              r = 2,
              b = 2,
              l = 2,
              unit = "cm"
          )
      )
plot_list(p, p1, ncol=1, tag_levels = 'A', tag_size = rel(2))
```

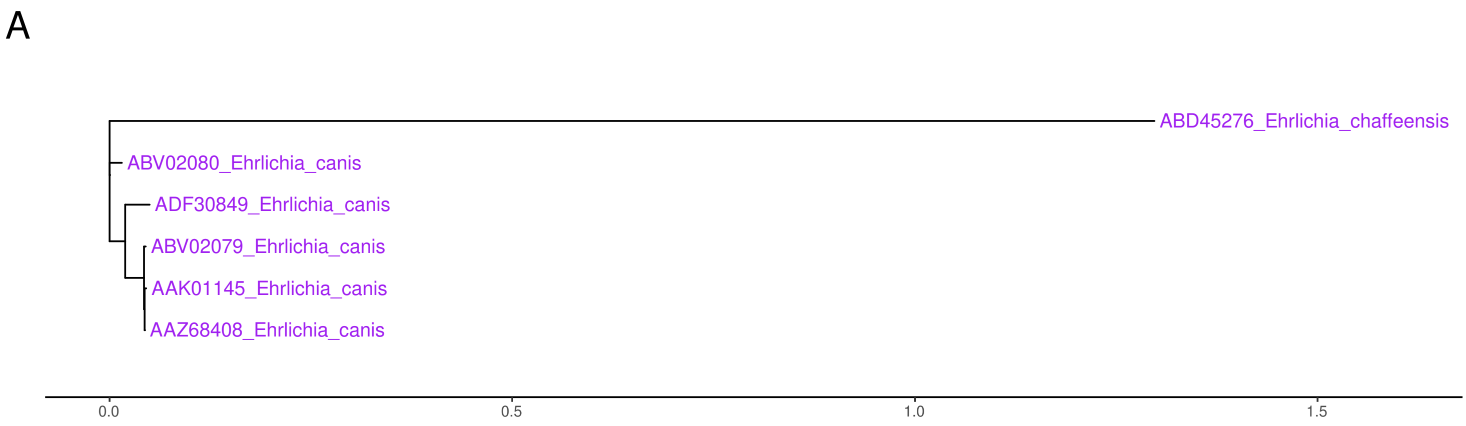

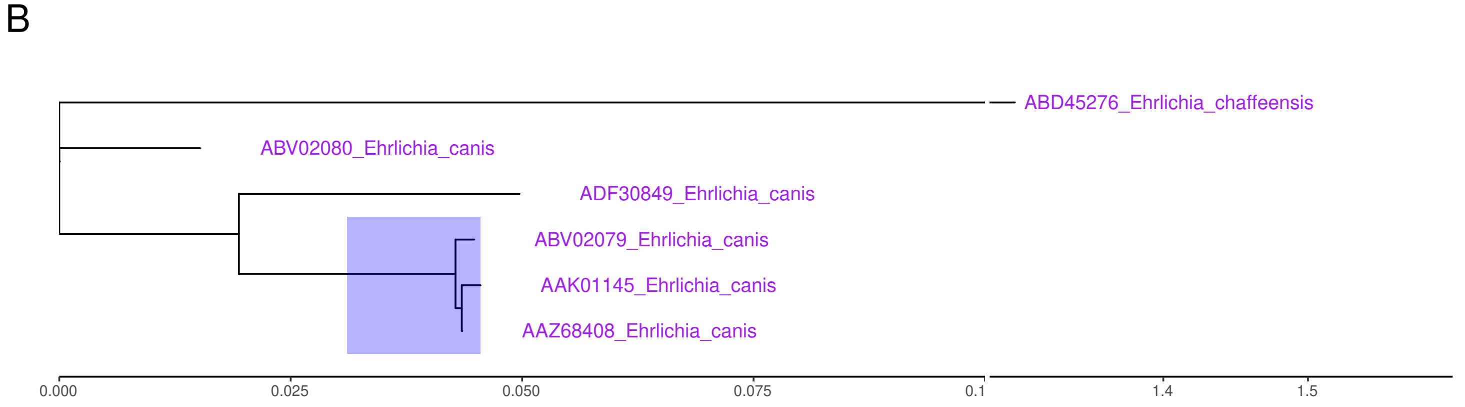

3 Example 3: Cut Manhattan plot to create more space for annotation

```
library("ggbreak")
library("aplot")
library("ggplot2")
library("dplyr")

snp <- read.table(file = "../data/GCST90007012_buildGRCh37.tsv",
                  header = T, fill = T)
head(snp)
```

```
##   variant_id p_value chromosome base_pair_location effect_allele    beta
## 1  rs3131972  0.3586          1             752721             A  10.880
## 2 rs11240777  0.4565          1             798959             A   8.063
## 3  rs4970383  0.3810          1             838555             A -10.080
## 4  rs4475691  0.8293          1             846808             T   3.277
## 5  rs7537756  0.2872          1             854250             G  14.660
## 6 rs13302982  0.8938          1             861808             A   1.374
##   standard_error
## 1          11.85
## 2          10.82
## 3          11.49
## 4          15.19
## 5          13.76
## 6          10.28
```

```
snp$chromosome <- factor(snp$chromosome, levels = sort(unique(snp$chromosome)))

p1 <- ggplot(
        data = snp,
        mapping = aes(
            x = chromosome,
            y = -log10(p_value),
```

```r
              color = chromosome)
      ) +
      geom_jitter() +
      geom_abline(
          intercept = 5,
          slope = 0,
          color = "grey",
          linetype = "dashed"
      ) +
      theme_classic() +
      xlab("Chromosomes") +
      theme(
          legend.position = "none",
          axis.title = element_text(size = 14),
          axis.text = element_text(size = 14)
      )

p2 <- p1 +
      scale_y_cut(
          breaks = 4.7,
          which = 2,
          scales = 0.2
      ) +
      geom_text(
          data=snp %>% filter(-log10(p_value) >= 5),
          mapping = aes(label = variant_id),
          nudge_x = 0.1,
          nudge_y = 0.1
      ) +
      expand_limits(x=c(0, 23), y=c(0, 7.5))

plot_list(p1, p2, ncol=1, tag_levels = 'A', tag_size = rel(2))
```

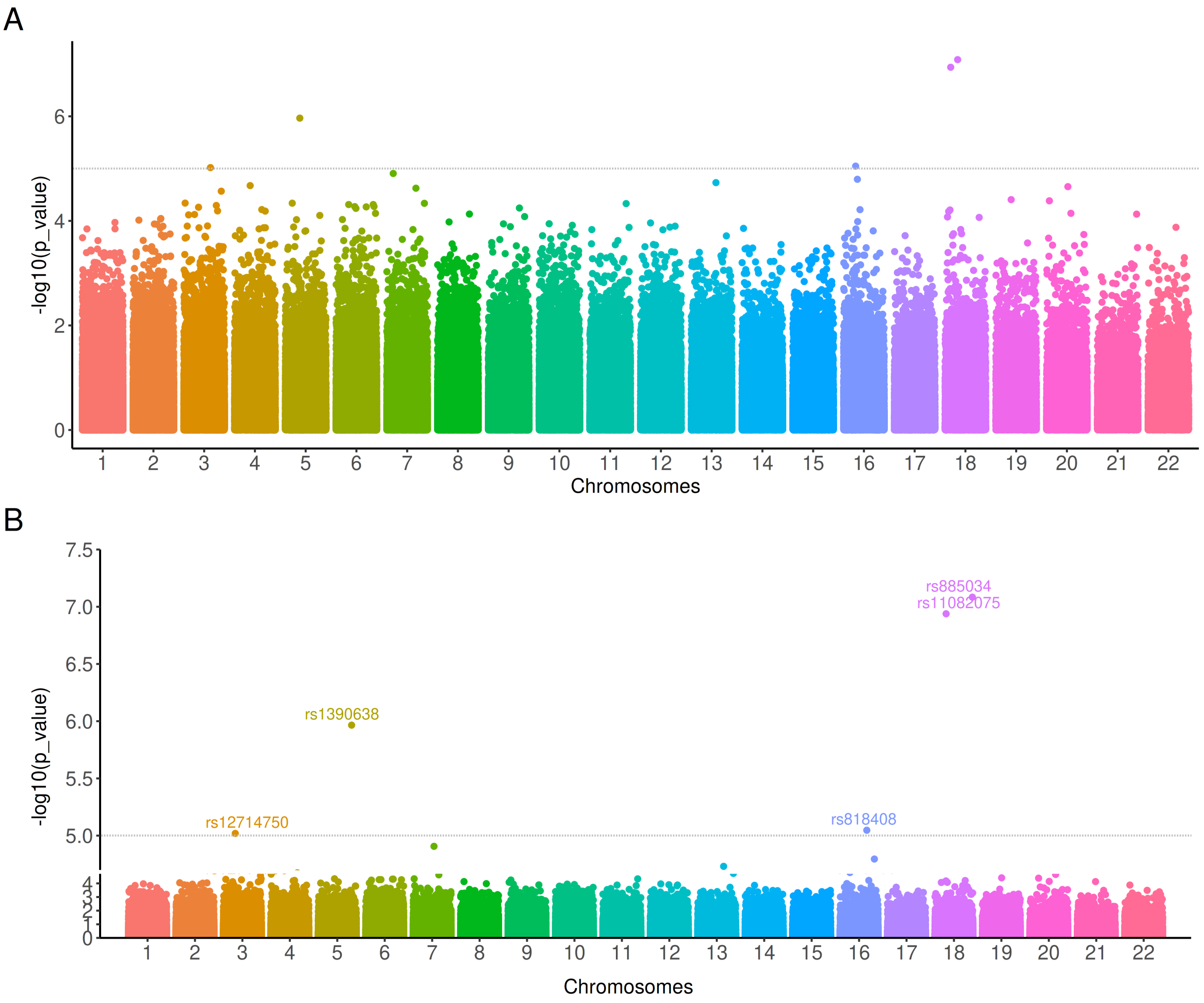

4 Example 4: Display discontinuous axis on a bar chart

```
library("ggprism")
library("ggbreak")
library("ggplot2")

data1 <- read.table(file = "../data/data1.txt",
                    header = TRUE, sep = "\t", dec = ".")
data1$Species.name <- factor(data1$Species.name,
                             levels = rev(unique(data1$Species.name)))
load("../data/sigdata.rda")

p1 <- ggplot(
          data = data1,
          mapping = aes(
              x = Species.name,
              weight = Mean,
              fill = group
          )
      ) +
```

```r
      geom_bar(position = 'dodge') +
      labs(y = 'Relative abundance(%)', x = NULL) +
      coord_flip() +
      theme_prism()

p2 <- ggplot(
          data = data1,
          mapping = aes(
              x = Species.name,
              weight = log10(Mean+1),
              fill = group
          )
      ) +
      geom_bar(position = 'dodge') +
      labs(y = 'log10(Relative abundance+1)', x = NULL) +
      coord_flip() +
      theme_prism()

p3 <- p1 +
      scale_y_break(c(32, 84), scales = 0.5, ticklabels=c(84, 85, 86)) +
      scale_y_break(c(3.5, 10), scales = 0.5, ticklabels = c(15, 25))

p4 <- p3 +
      geom_text(
          data = sigdata,
          mapping = aes(
              x = Species,
              y = Mean,
              label = sig
          ),
          vjust=-0.1
      )

plot_list(p1, p2, p3, p4, byrow=T, tag_levels = 'A', tag_size = rel(2))
```

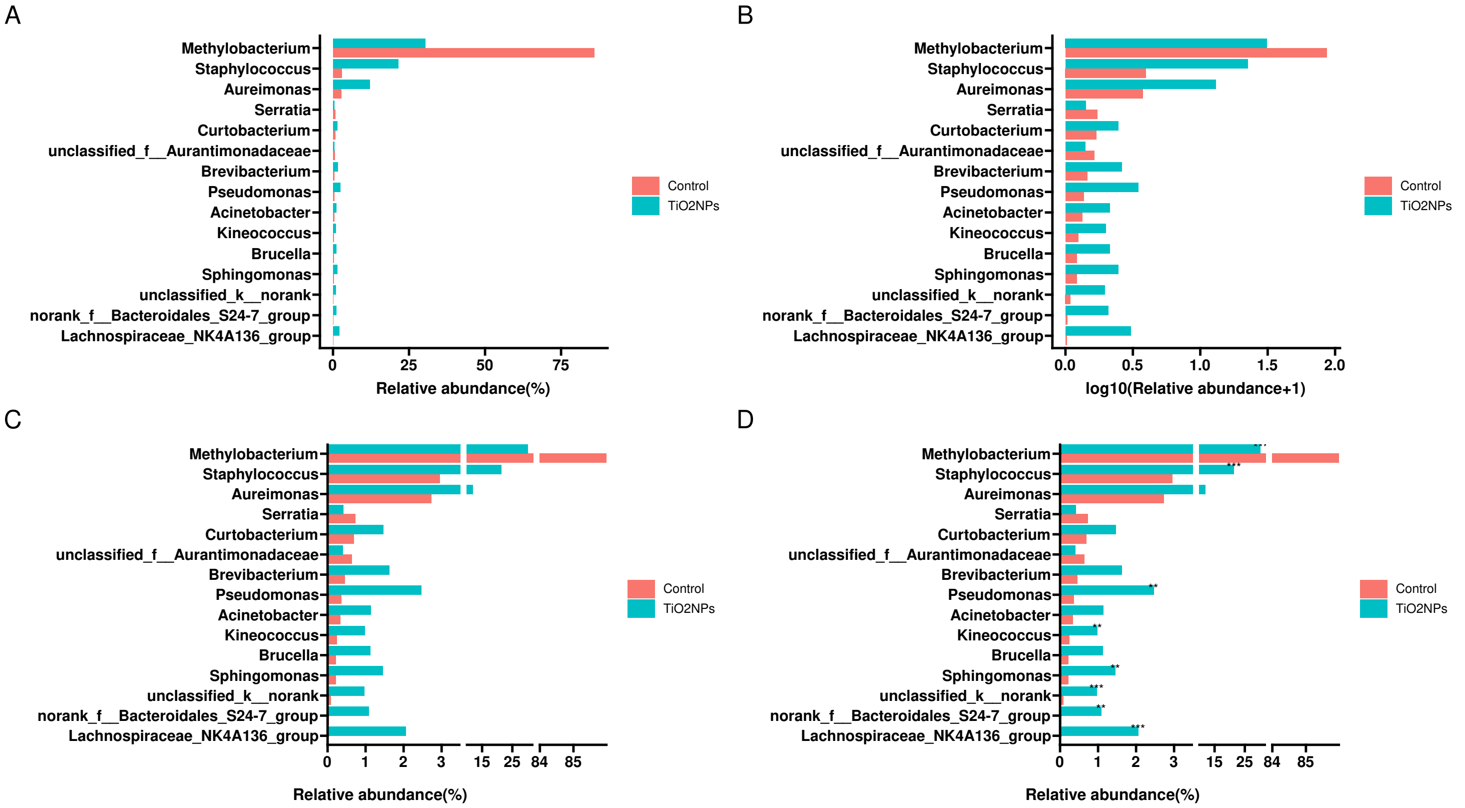
A
B
C
D
Methylobacterium
Staphylococcus
Aureimonas
Serratia
Curtobacterium
unclassified_f__Aurantimonadaceae
Brevibacterium
Pseudomonas
Acinetobacter
Kineococcus
Brucella
Sphingomonas
unclassified_k__norank
norank_f__Bacteroidales_S24-7_group
Lachnospiraceae_NK4A136_group
Control
TiO2NPs
Relative abundance(%)
log10(Relative abundance+1)
